# Wall Motion Score Index Predicts Persistent Moderate or Severe Secondary Mitral Regurgitation and its Prognostic Role in Patients Undergoing Percutaneous Coronary Intervention

**DOI:** 10.31083/j.rcm2409256

**Published:** 2023-09-18

**Authors:** Linfang Qiao, Haozhang Huang, Jiulin Liu, Congzhuo Jia, Yibo He, Sijia Yu, Hongyu Lu, Ziyou Zhou, Tian Chang, Shiqun Chen, Ning Tan, Jin Liu, Yong Liu, Jiyan Chen

**Affiliations:** ^1^The Second School of Clinical Medicine, Southern Medical University, 510515 Guangzhou, Guangdong, China; ^2^Department of Cardiology, Guangdong Cardiovascular Institute, Guangdong Provincial People's Hospital, Guangdong Academy of Medical Sciences, 510080 Guangzhou, Guangdong, China; ^3^Department of Guangdong Provincial Key Laboratory of Coronary Heart Disease Prevention, Guangdong Cardiovascular Institute, Guangdong Provincial People's Hospital, Guangdong Academy of Medical Sciences, 510080 Guangzhou, Guangdong, China; ^4^Guangdong Provincial People's Hospital, School of Medicine, South China University of Technology, 510100 Guangzhou, Guangdong, China

**Keywords:** persistent mitral regurgitation, coronary artery disease, percutaneous coronary intervention, wall motion score index, prognosis

## Abstract

**Background:**

Patients with secondary mitral regurgitation (sMR) often present with greater mortality and comorbidity, which may be predicted by some risk factors. This study was designed to investigate the prognostic meaning of the echocardiographically detected wall motion score index (WMSI) in coronary artery disease (CAD) patients with moderate or severe baseline sMR who underwent percutaneous coronary intervention (PCI) therapy.

**Methods:**

The present study was a multi-center and prospective cohort of consecutive CAD patients with baseline moderate or severe sMR who underwent PCI. All underwent echocardiography at baseline and at follow-up after PCI to assess sMR and WMSI. The primary endpoint was the persistence of moderate or severe sMR after the second echocardiographic measurement. Logistic and Cox proportional hazards models were constructed for the primary (persistent moderate or severe sMR) and secondary (worsening heart failure [HF]; all-cause mortality; cardiovascular-specific mortality; and major adverse cardiovascular events [MACE]) endpoints.

**Results:**

Among 920 participants, 483 had WMSI values of ≥1.47, and 437 were less. Of all the participants, 366 (39.8%) continued to have moderate or severe sMR after the second echocardiogram measurement. After full adjustment for confounders, elevated WMSI after PCI was independently associated with the primary endpoint during 3–12 month follow-up. Similarly, elevated WMSI was associated with increased risk of worsening HF, all-cause mortality, cardiovascular-specific mortality, and MACE.

**Conclusions:**

Persistent moderate or severe sMR is common (approximately 40%) in PCI patients. Elevated WMSI in CAD patients after PCI is a predictor of persistent moderate or severe sMR and has independent negative prognostic value. Patients with CAD and sMR should be monitored for WMSI to identify those at higher risk of mortality and comorbidity.

## 1. Introduction 

Secondary mitral regurgitation (sMR) is a frequent complication in patients with coronary artery disease (CAD), and results in greater mortality and comorbidity [1]. Percutaneous coronary intervention (PCI) can reduce reflux of sMR in the subsequent follow-up [2, 3, 4]. However, up to 30% of patients with moderate or severe sMR still have residual significant sMR after PCI; as a result, further adverse prognosis likely ensues [5].

Numerous clinical studies have reported trajectory problems in the changes of sMR after PCI [6, 7] and have identified risk factors for the progression of sMR, including significant left ventricular dilation, systolic dysfunction, and myocardial scar burden [8, 9, 10]. Semiquantitative assessment of regional systolic function using wall motion score index (WMSI) might be an alternative to left ventricular ejection fraction (LVEF) for the assessment of left ventricular systolic function, and some studies have indicated that the predictive value of WMSI for prognosis is greater than that of LVEF [11, 12, 13]. Increased WMSI could be considered a predictor of moderate or severe sMR [14]. However, the clinical impact of the WMSI on residual significant sMR in baseline moderate or severe cases has not been sufficiently characterized.

Therefore, in the present study, we analyzed the relationship between WMSI and persistent moderate or severe sMR and the prognostic meaning of the extent of echocardiographically detected WMSI in a consecutive series of patients with moderate or severe baseline sMR who underwent PCI therapy.

## 2. Materials and Methods

### 2.1 Study Sample 

This cohort study examined data from the Cardiorenal Improvement-II, a prospective and observational multi-center database of patients enrolled between January 2007 and December 2020 from five large tertiary hospitals in southern China. In order to diagnose CAD, the 10th Revision of the Codes of the International Classification of Diseases was utilized. The indication of PCI or coronary angiography included signs or symptoms of ischemia, elevated cardiac enzymes, or diagnostic electrocardiogram, performed in compliance with standard clinical practice guidelines [15, 16].

**Fig. 1.**
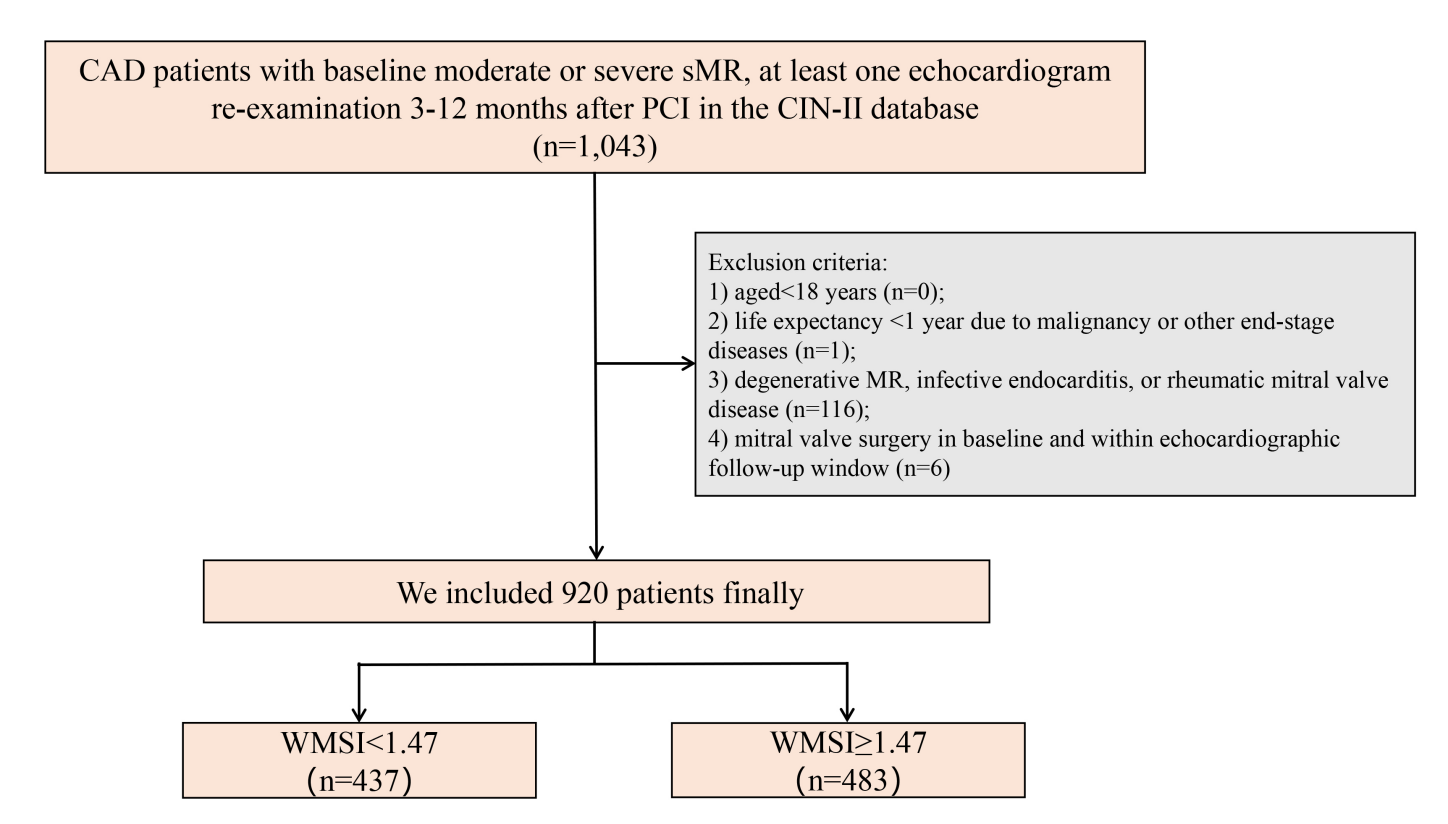
**Flowchart of the study**. CAD, coronary artery disease; sMR, secondary mitral regurgitation; PCI, percutaneous coronary intervention; WMSI, wall motion score index; CIN-II, Cardiorenal Improvement II; MR, mitral regurgitation.

Data from 1043 CAD patients from the Cardiorenal Improvement-II (CIN-II) database with baseline moderate or severe sMR undergoing PCI upon admission and had at least one echocardiographic re-examination 3 month–1 year post-PCI, were initially examined. Exclusion criteria were: (a) age <18 year; (b) life expectancy <1 year due to end-stage diseases; (c) degenerative MR, infective endocarditis, or rheumatic mitral valve disease; and (d) mitral valve surgery in baseline and within echocardiographic follow-up window. Therefore, 920 patients were finally included for analysis (Fig. 1).

The Ethics Committee of the Guangdong Provincial People’s Hospital approved the study (Approval No. GDREC2019555H[R1]). It was conducted in accordance with the principles of the Declaration of Helsinki. As of June 1, 2022, all patients were followed up by telephone and by the Guangdong Center for Disease Control and Prevention (CDC), according to the ID numbers of the patients, to obtain survival data. All participants provided oral informed consent by telephone.

### 2.2 Echocardiographic Assessment 

The echocardiographic data were obtained by trained sonographers and analyzed by experienced cardiologists at the Echocardiography Reading Center, located at the Guangdong Provincial People’s Hospital. Post-PCI instructions advised all patients of the required examination schedule (at least one echocardiographic exam 3–12 month after PCI). If patients had undergone several echocardiographic examinations over time, we used the latest post-PCI echocardiogram to assess the severity of MR.

The presence of MR was determined on the first echocardiographic examination, generally within 48 h of admission. (A small number of echocardiographic examinations were assessed after the procedure because of emergency PCI). The echocardiographic report was used to determine the presence and severity of MR and classified as none, mild, moderate, moderately severe, or severe. The classification was performed through a visual assessment integrating Doppler data from multiple acoustic windows, including qualitative and semi-quantitative methods. The definition of MR was established beforehand by mitral valve morphology data-field descriptors included in the echocardiographic database. The mitral valve morphologic descriptors included abnormal, myxomatous, flail, prolapsed, or thickened valves, and degenerative MR was diagnosed based on these descriptors. MR was classified as secondary when there was no intrinsic mitral valve leaflet disease. Persistent moderate or severe sMR was defined as baseline moderate or severe which was then still present as moderate or severe during follow-up.

The echocardiography-derived WMSI was used to evaluate regional left ventricular function. The segmentation of the left ventricle followed a 17-segment model as recommended by the American Society for Echocardiography [17]. The function of each segment was confirmed in multiple views and recorded on videotape. Two experienced observers, who were not aware of the clinical data, evaluated the echocardiographic examination. Segments were scored using the following criteria: normal or hyperkinesis = 1, hypokinesis = 2, akinesis = 3, and dyskinesis (or aneurysmatic) = 4. The WMSI was obtained by dividing the sum of all scores by the number of segments visualized.

### 2.3 Study Endpoint 

The primary endpoint of the study was the persistence of moderate or severe sMR. Secondary endpoints included worsening heart failure (HF) after the second echocardiogram measurement, all-cause mortality, cardiovascular-specific mortality, and major adverse cardiovascular events (MACE). Worsening HF was defined as unplanned rehospitalization or unscheduled physician office/emergency visit due to a primary diagnosis of HF. MACE was defined as cardiovascular-specific mortality, acute myocardial infarction, or stroke. Cardiovascular-specific mortality was identified by using the underlying cause-of-death 10th Revision Codes of the International Classification of Diseases (ICD-10).

### 2.4 Statistical Analysis 

For statistical analysis, our study sample was divided into two groups based on the median WMSI (median = 1.47). Descriptive statistics are reported as the mean (standard deviation [SD]), median (interquartile range, [IQR]), or number and percentage when appropriate. The Chi-square test was used to compare differences between categorical variables. The independent samples Student’s *t*-test was used to compare continuous variables with normal distribution, and the Mann-Whitney U test was used to compare continuous variables without normal distribution.

Endpoints were assessed using the Kaplan-Meier method and were compared using the log-rank test. The independent association between WMSI and outcomes was assessed with logistic and Cox regression models and expressed as the adjusted odds ratio (OR) or hazard ratio (HR) with 95% confidence interval (CI). Covariates were chosen based on prior literature and clinical experience [18, 19, 20, 21]. This included age, gender, smoking history, hypertension, diabetes mellitus, anemia, chronic kidney disease (CKD), atrial fibrillation, and acute myocardial infarction. Similar models were used for the secondary endpoints. We also performed a subgroup analysis among four prespecified subgroups — gender, age, acute coronary syndrome (ACS) [Yes or No], and CKD [Yes or No] — to assess the impact of WMSI on persistent moderate or severe sMR, and then calculated the *p* value to assess the relationship between the endpoints and subgroups.

All *p*-values were 2 sided, with *p*-values < 0.05 statistically significant. All models used met the proportional hazards (PH) assumption. Statistical analyses were performed using R ver. 4.1.3 (R Institute for Statistical Computing, Vienna, Austria).

## 3. Results

### 3.1 Baseline Characteristics 

A total of 920 CAD patients who underwent PCI with baseline moderate or severe sMR, and who presented with remeasurements of sMR severity from 3 month–1 year, were included in the analysis. There were 483 patients (53%) with WMSI values ≥1.47 (high-score group), and 437 patients (47%) with WMSI values <1.47 (low-score group). Of those, 366 (39.8%) had persisting moderate or severe sMR after the second echocardiogram measurement. The mean age was 64.1 ± 11.0 year. Patients in the low-score group were older, and males accounted for 79.6% of all patients (n = 732). Some high-risk comorbidities were more common in the high-score group, such as CKD (42.4% vs 30.9%, *p <* 0.001), moderate or severe pulmonary arterial hypertension [22] (PAH) (31.3% vs 19.9%, *p* < 0.001), and congestive heart failure (CHF) (53.4% vs 36.2%, *p* < 0.001). Moreover, the high-score group patients had a larger left atrial (LA) size, left ventricular end-diastolic dimension, left ventricular end-systolic dimension, and a lower LVEF, with more severe calcification, and were more likely to require complex PCI. The greater use of mineralocorticoid receptor antagonists and loop diuretic medications in the high-score group was consistent with the increased risk of CHF in that group. The characteristics of the patients at baseline are shown in Tables 1,2.

**Table 1.** **Baseline clinical characteristics**.

Characteristic	Overall	WMSI <1.47	WMSI ≥1.47	*p*-value
	N = 920	N = 437	N = 483	
Male	732 (79.6)	326 (74.6)	406 (84.1)	**0.001**
Age, yrs	64.1 (11.0)	65.3 (11.5)	63.0 (10.4)	**0.001**
BMI, Kg/$m^{2}$	23.75 (3.42)	23.92 (3.26)	23.58 (3.57)	0.178
HR, bmp	82.14 (17.35)	78.92 (15.84)	85.05 (18.15)	< **0.001**
SBP, mmHg	128.12 (22.78)	131.39 (23.18)	125.16 (22.01)	< **0.001**
DBP, mmHg	75.64 (12.85)	75.07 (12.81)	76.16 (12.88)	0.202
History of smoke				0.485
Never	566 (61.5)	277 (63.4)	289 (59.8)	
Cessation	139 (15.1)	65 (14.9)	74 (15.3)	
Current	215 (23.4)	95 (21.7)	120 (24.8)	
Cardiac function				< **0.001**
I	237 (25.8)	158 (36.2)	79 (16.4)	
II	391 (42.5)	182 (41.6)	209 (43.3)	
III	222 (24.1)	77 (17.6)	145 (30.0)	
IV	70 (7.6)	20 (4.6)	50 (10.4)	
Anemia	402 (43.7)	193 (44.2)	209 (43.3)	0.837
Congestive heart failure	416 (45.2)	158 (36.2)	258 (53.4)	< **0.001**
Diabetes	630 (68.5)	289 (66.1)	341 (70.6)	0.166
Chronic kidney disease	340 (37.0)	135 (30.9)	205 (42.4)	< **0.001**
Hypertension	551 (59.9)	286 (65.4)	265 (54.9)	**0.001**
Hyperlipidemia	689 (74.9)	318 (72.8)	371 (76.8)	0.182
Atrial fibrillation	104 (11.3)	58 (13.3)	46 (9.5)	0.091
COPD	29 (3.2)	9 (2.1)	20 (4.1)	0.106
Stroke	39 (4.2)	20 (4.6)	19 (3.9)	0.749
History of PCI	119 (12.9)	57 (13.0)	62 (12.8)	>0.99
History of AMI	99 (10.8)	44 (10.1)	55 (11.4)	0.591
Clinical presentation				
AMI	343 (37.3)	177 (40.5)	166 (34.4)	0.064
STEMI	227 (24.7)	113 (25.9)	114 (23.6)	0.474
NSTEMI	116 (12.6)	64 (14.6)	52 (10.8)	0.095
Chronic coronary syndrome	267 (29.0)	137 (31.4)	130 (26.9)	0.159
Baseline laboratory				
LDL-C, mmol/L	2.88 (1.10)	2.93 (1.15)	2.83 (1.06)	0.182
HDL-C, mmol/L	0.97 (0.27)	0.99 (0.27)	0.95 (0.28)	0.053
eGFR, mL/min/1.73 $m^{2}$	69.65 (25.97)	73.01 (27.39)	66.60 (24.24)	< **0.001**
Albumin, g/L	34.96 (4.60)	35.25 (4.77)	34.69 (4.42)	0.067
NT-proBNP, ng/L	2077.00 [902.35, 4830.00]	1423.00 [548.72, 3473.75]	2652.00 [1227.00, 5574.00]	< **0.001**
hs-cTnT, ng/L	0.71 [0.22, 7.46]	0.86 [0.17, 7.32]	0.69 [0.26, 7.60]	0.495
Baseline Procedural characteristics				
Emergent PCI	264 (28.7)	136 (31.1)	128 (26.5)	0.140
Radial artery access	752 (81.7)	377 (86.3)	375 (77.6)	**0.001**
Multivessel disease	787 (85.5)	371 (84.9)	416 (86.1)	0.662
Culprit vessel in STEMI				**0.011**
Left main coronary artery	7 (2.8)	1 (0.8)	6 (4.8)	0.434
LAD	115 (46.6)	48 (39.0)	67 (54.0)	0.890
LCX	41 (16.6)	23 (18.7)	18 (14.5)	0.112
RCA	84 (34.0)	51 (41.5)	33 (26.6)	0.997
Lesion morphology*				
Moderate/severe calcification	386 (42.0)	154 (35.2)	232 (48.0)	< **0.001**
Thrombotic	95 (10.3)	45 (10.3)	50 (10.4)	>0.99
Bifurcation	353 (38.4)	153 (35.0)	200 (41.4)	0.054
Total occlusion	519 (56.4)	211 (48.3)	308 (63.8)	< **0.001**
Multivessel CAD	787 (85.5)	371 (84.9)	416 (86.1)	0.662
Number of vessels treated	1.45 (0.68)	1.40 (0.68)	1.49 (0.68)	0.063
Left main coronary artery treated	92 (10.0)	38 (8.7)	54 (11.2)	0.252
LAD treated	515 (56.0)	223 (51.0)	292 (60.5)	**0.005**
LCX treated	298 (32.4)	133 (30.4)	165 (34.2)	0.256
RCA treated	426 (46.3)	218 (49.9)	208 (43.1)	**0.045**
Number of stents	1.91 (1.18)	1.79 (1.12)	2.02 (1.22)	**0.002**
Minimum stent diameter, mm	2.69 (0.76)	2.72 (0.80)	2.67 (0.72)	0.326
Stent length, mm**†**	52.27 (36.32)	47.97 (34.46)	56.16 (37.54)	**0.001**
Complex PCI**‡**	436 (47.4)	180 (41.2)	256 (53.0)	< **0.001**
Complete ${\mathrm{PCI}}^{\$}$	395 (42.9)	200 (45.8)	195 (40.4)	0.113
Discharge prescription				
RAAS inhibitor	616 (67.0)	296 (67.7)	320 (66.3)	0.684
Beta-blockers	780 (84.8)	358 (81.9)	422 (87.4)	**0.027**
Calcium channel blockers	143 (15.5)	84 (19.2)	59 (12.2)	**0.005**
Statin	854 (92.8)	401 (91.8)	453 (93.8)	0.288
Aspirin	870 (94.6)	422 (96.6)	448 (92.8)	**0.016**
Clopidogrel	807 (87.7)	382 (87.4)	425 (88.0)	0.868
Loop diuretic	459 (49.9)	154 (35.2)	305 (63.1)	< **0.001**
MRA	465 (50.5)	152 (34.8)	313 (64.8)	< **0.001**

Median (interquartile range). **Bold** indicates statistical significance. *Lesion morphology assessed by operators. †Stent length calculated by operators. ‡Complex PCI was defined as any of the following: ≥3 vessels treated, ≥3 lesions treated, lesion length >60 mm, bifurcation with 2 stents implanted, or chronic total occlusion as target lesion. $Complete PCI was defined as the following: the stenosis of ≥50% in the left main coronary artery for treatment, the stenosis of ≥70% in LAD, LCX, or RCA for treatment. Abbreviation: yrs, years; AMI, acute myocardial infarction; COPD, chronic obstructive pulmonary disease; DBP, diastolic blood pressure; eGFR, estimated glomerular filtration rate; HR, heart rate; HDL-C, high-density lipoprotein cholesterol; hs-cTnT, Hypersensitive troponin T; LDL-C, low-density lipoprotein cholesterol; LAD, left anterior descending coronary artery; LCX, left circumflex coronary artery; MRA, mineralocorticoid recept antagonist; NSTEMI, non-ST-segment elevation myocardial infarction; NT-proBNP, N-terminal pro brain natriuretic peptide; PCI, percutaneous coronary intervention; RAAS inhibitor, renin-angiotensin-aldosterone system inhibitor; RCA, right coronary artery; SBP, systolic blood pressure; STEMI, ST-segment elevation myocardial infarction; WMSI, wall motion score index; BMI, body mass index; CAD, coronary artery disease.

**Table 2.** **Baseline Characteristics of echocardiography**.

Characteristic	Overall	WMSI <1.47	WMSI >1.47	*p*-value
	N = 920	N = 437	N = 483	
LVEF, %	43.82 (13.67)	53.00 (11.34)	35.53 (9.74)	< **0.001**
LVEDD, mm	56.64 (8.54)	52.65 (7.67)	60.26 (7.63)	< **0.001**
LVESD, mm	43.30 (10.66)	37.38 (9.04)	48.67 (9.07)	< **0.001**
Left atrial, mm	41.23 (6.34)	39.80 (6.12)	42.54 (6.25)	< **0.001**
LVPWT, mm	9.50 (1.96)	9.83 (2.03)	9.19 (1.86)	< **0.001**
IVS, mm	10.11 (2.39)	10.52 (2.43)	9.74 (2.29)	< **0.001**
E peak of mitral valve, m/s	0.90 (0.26)	0.90 (0.26)	0.90 (0.27)	0.654
A peak of mitral valve, m/s	0.74 (0.28)	0.78 (0.27)	0.70 (0.27)	< **0.001**
E/A ratio of mitral valve	1.42 (0.79)	1.32 (0.72)	1.52 (0.84)	< **0.001**
PAH	238 (25.9)	87 (19.9)	151 (31.3)	< **0.001**
WMSI total†	25.97 (6.29)	20.52 (2.37)	30.90 (4.35)	< **0.001**

Values are mean ± SD or n (%). **Bold** indicates statistical significance. †Using a standard transthoracic echocardiography sequence, each myocardial segment in 17 segment model is assigned a score from 1 to 4. Abbreviation: IVS, interventricular septum; LVEF, left ventricular ejection fraction; LVEDD, left ventricular end-diastolic dimension; LVESD, left ventricular end-systolic dimension; LVPWT, left ventricular posterior wall thickness; PAH, pulmonary arterial hypertension; WMSI, wall motion score index.

### 3.2 Primary Outcome

The median interval between the baseline and follow-up echocardiography was 6.5 month. The unadjusted odds ratio obtained by logistic proportional regression is shown in Table 3. After adjusting for confounding factors, elevated WMSI after PCI was found to be a significant independent predictor of persistent moderate or severe sMR (OR: 1.53; 95% CI: 1.15–2.03; *p* = 0.003) compared to their counterparts with WMSI values <1.47, in multivariable logistic regression analyses (Table 3).

**Table 3.** **Primary and secondary outcomes**.

Outcomes	Total	WMSI <1.47	WMSI ≥1.47	Unadjusted		${\mathrm{Adjusted}}^{*}$	
	(N = 920)	(N = 437)	(N = 483)				
Primary outcome				OR (95%)	*p* value	OR (95%)	*p* value
Persistent moderate or severe sMR	366 (39.8%)	157 (17.1%)	209 (22.7%)	1.36 (1.04–1.78)	0.023	1.53 (1.15–2.03)	0.003
Secondary outcomes (5-year)				HR (95%)	*p* value	HR (95%)	*p* value
Worsening HF	133 (14.5%)	46 (5.0%)	87 (9.5%)	1.79 (1.25–2.56)	0.001	1.94 (1.34–2.80)	<0.001
All-cause death	184 (20.0%)	73 (7.9%)	111 (12.1%)	1.39 (1.04–1.87)	0.027	1.46 (1.07–1.98)	0.016
Cardiovascular-specific death	136 (14.8%)	54 (5.9%)	82 (8.9%)	1.39 (0.99–1.96)	0.059	1.47 (1.03–2.09)	0.035
MACE	192 (20.9%)	78 (8.5%)	114 (12.4%)	1.34 (1.01–1.79)	0.046	1.41 (1.05–1.90)	0.024

The independent association between WMSI and outcomes was assessed with logistic (primary outcome) and Cox regression (secondary outcomes) model and expressed as the adjusted OR or HR with 95% confidence interval. *Adjusted for age (as a continuous variable), gender, smoking history, hypertension, diabetes mellitus, anemia, chronic kidney disease, acute myocardial infarction, atrial fibrillation. WMSI, wall motion score index; HF, heart failure; sMR, secondary mitral regurgitation; MACE, major adverse cardiovascular events; OR, odds ratio; HR, hazard ratio.

### 3.3 Secondary Outcomes

**Fig. 2.**
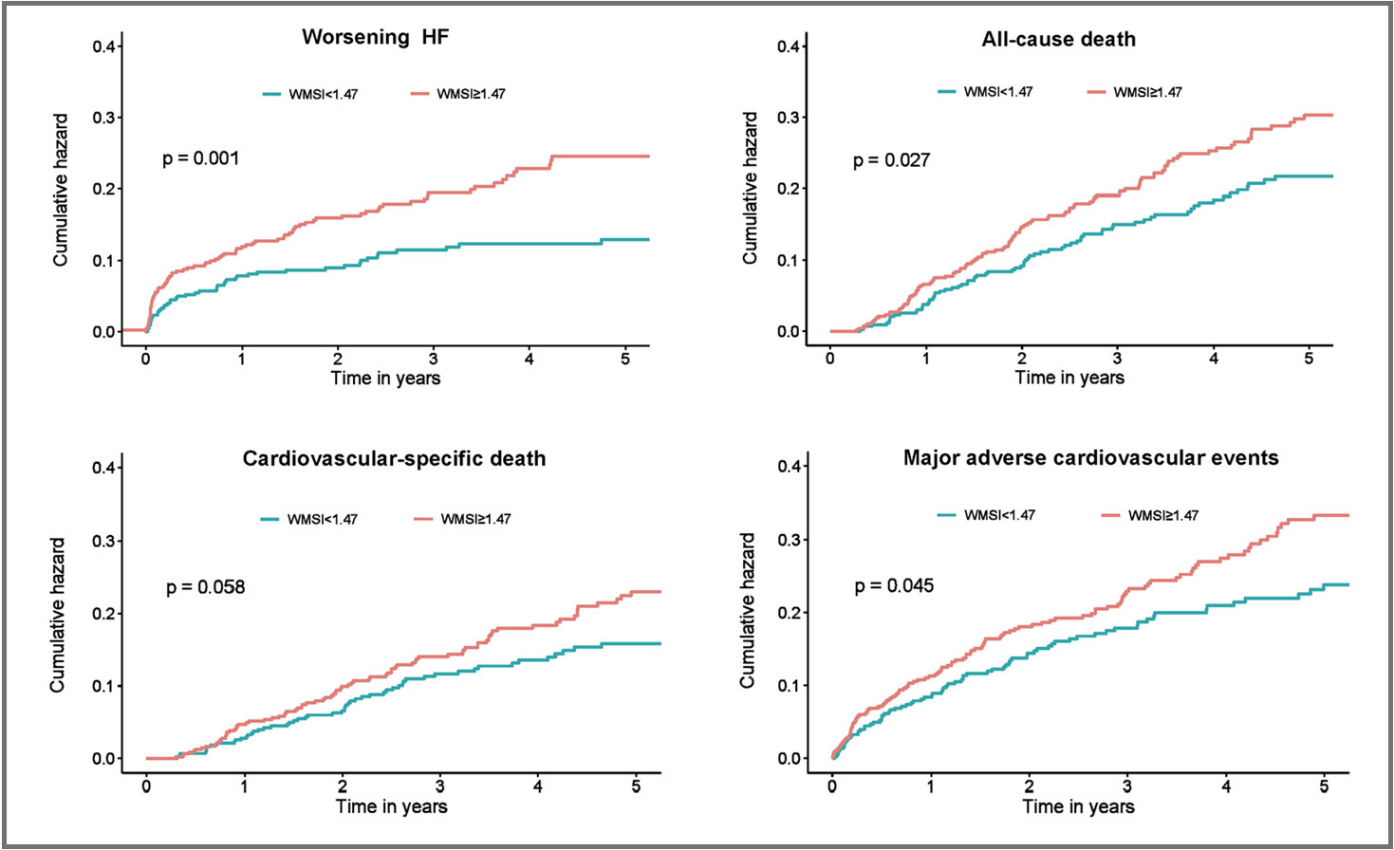
**Secondary Outcomes of worsening HF, all-cause death, cardiovascular-specific death, or major adverse cardiovascular events**. Shown are Kaplan–Meier estimates of the cumulative incidence of worsening HF, all-cause death, cardiovascular-specific death, or major adverse cardiovascular events during 5 year follow-up. HF, heart failure; WMSI, wall motion score index.

During a median follow-up of 2.8 year (IQR: 1.8–3.7 year), 184 (20.0%) patients died; cardiovascular mortality accounted for 136 (73.9%) of the deaths. Among all patients, there were 192 (20.9%) patients with MACE and 133 (14.5%) patients with worsening HF. The relationship between WMSI and secondary endpoints showed a similar pattern to that of the primary endpoint. After full adjustment for confounders, elevated WMSI also proved to be an independent predictor of worsening HF (HR: 1.94; 95% CI: 1.34–2.80; *p* < 0.001), all-cause mortality (HR: 1.46; 95% CI: 1.07–1.98; *p* = 0.016), cardiovascular-specific mortality (HR: 1.47; 95% CI: 1.03–2.09; *p* = 0.035), and MACE (HR: 1.41; 95% CI: 1.05–1.90; *p* = 0.024) (Table 3). The Kaplan–Meier survival curve revealed the same survival outcome except for cardiovascular-specific mortality (Fig. 2).

**Fig. 3.**
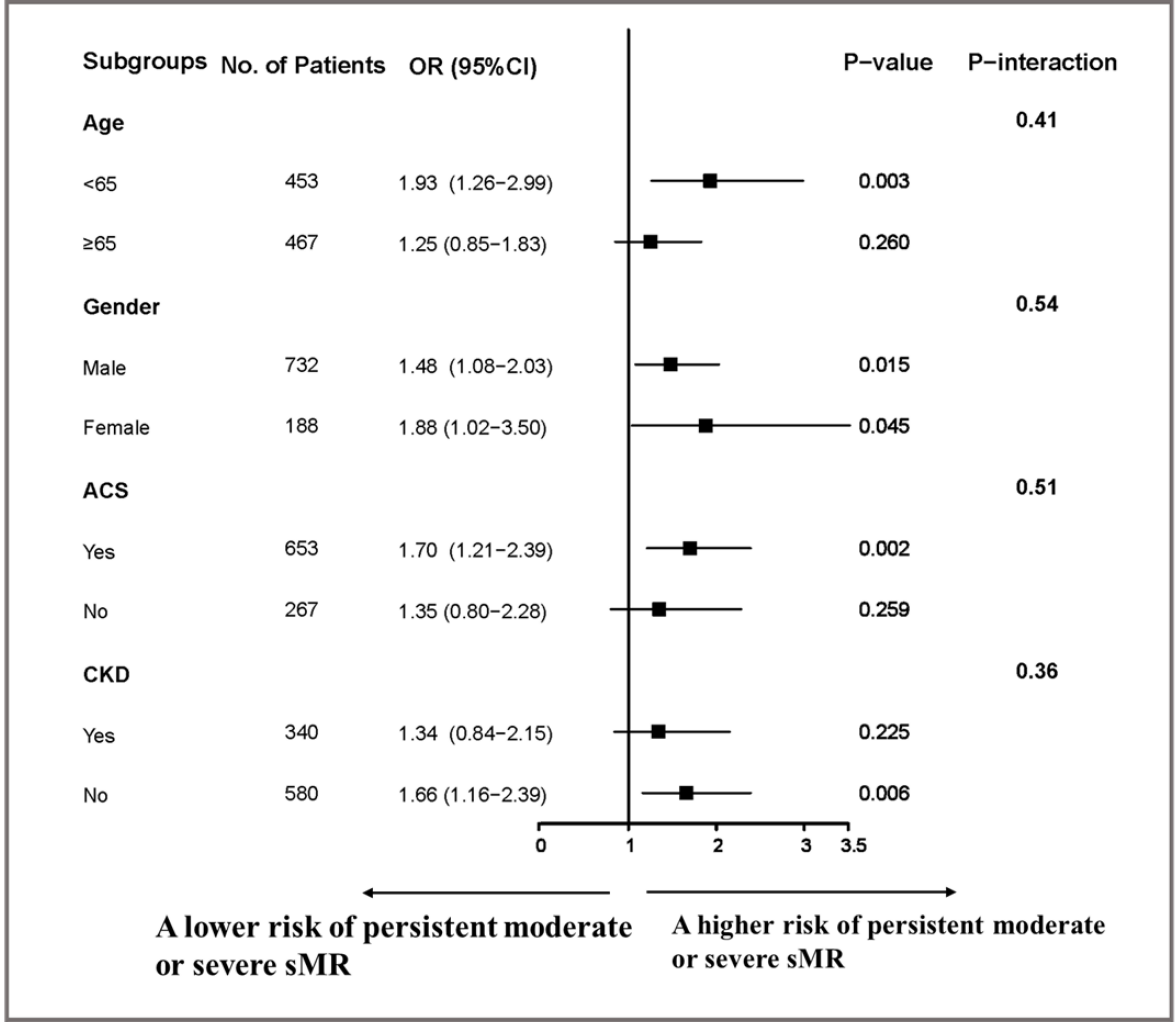
**Subgroup analyses of the persistent moderate or severe sMR **. Shown is a forest plot of odds ratio for persistent moderate or severe sMR event according to prespecified subgroups. ACS, acute coronary syndrome; CKD, chronic kidney disease; sMR, secondary mitral regurgitation; OR, odds ratio.

We conducted subgroup analyses to explore potential heterogeneity in the association between WMSI and the risk of persistent moderate or severe sMR. The results revealed consistent positive associations in several subgroups, while no significant associations were observed in the Age ≥65, Non-ACS, or Presence of CKD subgroups. These negative findings may be due to the limited sample sizes and insufficient statistical power, as the odds ratios in these subgroups were larger than one, and the *p* values for interaction were greater than 0.05 (Fig. 3). Overall, these findings suggest that the predictive value of WMSI for persistent sMR may vary across different patient subgroups, and further studies with larger sample sizes are warranted to confirm our findings.

## 4. Discussion

In this cohort, we found persistent moderate or severe sMR in more than 1/3 of the post-PCI patients. Elevated WMSI was independently associated with persistent moderate or severe sMR, conferring a 1.5-fold increased risk among CAD patients with baseline moderate or severe sMR at follow-up. The extent of echocardiographically detected WMSI before discharge might be an important predictor of comorbidity and mortality among these patients.

Epidemiological data suggest that moderate or severe sMR is a frequent cause of hospital admission, including readmission for heart failure, HF-related hospitalization, and all-cause hospitalization, which poses a significant societal burden [23, 24, 25]. In the chronic phase after myocardial infarction (MI), the presence of baseline sMR is associated with increased mortality, and the risk of mortality is directly related to the severity of sMR. Notably, sMR progression is also an independent predictor of poor outcomes. sMR progression is significantly and independently associated with more advanced left ventricular (LV) dilation and more extensive MI. Moreover, sMR progression provides additional risk stratification for patients with significant sMR at baseline. Patients with severe sMR but no significant sMR progression over time demonstrated significantly improved survival compared to those with severe sMR and continued progressive sMR [26, 27]. Revascularization has shown reliable improvement in sMR [28]. Many studies indicated that PCI is known to improve overall outcomes, can reduce the area of myocardial ischemia and reflux of sMR in subsequent follow-ups [2, 3]. One study demonstrated that in patients with severe sMR and CAD, PCI alone improved sMR in approximately 1/3 of patients (36%), and in at least 3/4 of these patients, the improvement was sustained [5]. However, sMR is known to be dynamic in nature: a proportion of patients show worsened sMR after an ischemic event or deterioration of HF even after accepting PCI, which could be easily overlooked by clinicians and researchers.

Some echocardiographic indicators, like end-systolic volume, have been shown to be predictors of worse outcomes, and have recently emerged as tools for predicting the progression of sMR [26]. Some studies have identified risk factors for progression of sMR, including significant LV dilation, systolic dysfunction, and myocardial scar burden [8, 9, 10]. Now, semiquantitative assessment of regional systolic function using WMSI is an alternative to LVEF for the assessment of left ventricular systolic function, and some studies have indicated that the predictive power of WMSI for prognosis is greater than that of LVEF [11, 12, 13].

Some previous studies have suggested that WMSI is superior to LVEF in predicting the combined endpoint of death, nonfatal reinfarction, unstable angina, and rehospitalization for CHF [12, 13]. Furthermore, LVEF may be almost normal, despite extensive regional wall motion abnormalities due to compensatory regional hyperkinesis [11, 29, 30]. Indeed, the left ventricle that undergoes post-infarction remodeling is a complex mixture of scar tissue (with varying degrees of transmurality) and residual myocardium with varying contractility. Traditional volume-based indices, such as left ventricular end-diastolic volume or LVEF, are inadequate in predicting outcomes since they depend on global ventricular measurements. Therefore, a more comprehensive screening tool is needed that accounts for the variability in function across different regions of the ventricle. In this regard, the WMSI holds promise as a reliable indicator since it can accurately reflect this information and provide a more nuanced assessment of ventricular function [31, 32].

There are many controversial findings in the literature regarding the precise mechanisms of sMR. Classically, significant ventricular remodeling and resultant apical displacement of the papillary muscles are thought to be the main contributors to sMR [33, 34, 35]. Post-ischemic LV remodeling is a gradual and continuous process. This process results in LV enlargement, thinning of the LV walls, increased wall stress, and progressive LV dysfunction. The LV distortion caused by post-ischemic LV remodeling, in which the LV becomes spherical rather than elliptical, can contribute to the development of ischemic mitral regurgitation (MR). This is due to changes in the dynamics of the mitral valve, which can result from papillary muscle dysfunction, mitral annulus dilation, and incomplete leaflet coaptation. Thus, the development of ischemic MR is influenced by the pathophysiological and mechanistic impact of LV distortion. It is crucial to effectively manage post-ischemic LV remodeling to prevent the progression of LV dysfunction and reduce the risk of developing ischemic MR [36, 37]. Kalra *et al*. [38] proposed a new mechanism of ischemic MR. It is based on the fact that the loss of wall thickening in the myocardial middle segments of the inferolateral and inferior walls reduced the interpapillary muscle distance, which tethered mitral leaflet edges and thus impaired their systolic closure independently of LV dilatation.

For patients with moderate or severe sMR undergoing PCI, employing targeted analyses for risk factors facilitated early identification. In addition, intervention before irreversible deterioration of sMR is warranted. An examination of WMSI can facilitate the prediction of persistent moderate or severe sMR and the prognosis of poor outcomes, suggesting the need for aggressive therapeutic interventions when coronary intervention by itself is not enough. In recent decades, several strategies have been developed, such as transcatheter mitral valve interventions (i.e., the Mitra-Clip procedure), in order to improve the reflux degree of moderate to severe sMR and reduce the risk of poor outcomes. However, it has not been widely used in the clinic. Two randomized controlled clinical trials have investigated the effects of MitraClip on patients with HF: the Percutaneous Repair with the MitraClip Device for Severe Functional/Secondary Mitral Regurgitation (MITRA-FR) trial, which examined the effects of percutaneous repair with the MitraClip device on severe functional/secondary mitral regurgitation, and the Cardiovascular Outcomes Assessment of the MitraClip Percutaneous Therapy for Heart Failure Patients with Functional Mitral Regurgitation (COAPT) trial, which assessed the cardiovascular outcomes of MitraClip percutaneous therapy in HF patients with functional mitral regurgitation. In both trials, patients were randomly assigned to receive either MitraClip plus guideline-directed medical therapy (GDMT) or GDMT alone. While MITRA-FR did not demonstrate any significant reduction in the primary endpoint of all-cause mortality or HF hospitalizations, the COAPT trial reported a significant decrease in HF hospitalizations (primary endpoint) as well as in mortality alone. Nonetheless, the effectiveness of transcatheter mitral valve interventions in certain adapted populations remains a contentious issue [39, 40, 41]. Further studies are needed to determine whether patients who are at higher risk of progressive sMR would benefit from mitral valve intervention.

There are several limitations to the present study. First, there are many etiologies for sMR. In our study, the main goal was post-PCI residual moderate or severe sMR, and it was necessary to study the related factors and prognosis in other residual significant sMR samples. Second, inherent in the observational nature of this study, there are likely significant residual unmeasured confounding factors for prognosis; our results should therefore be considered hypothesis-generating. Furthermore, echocardiography and WMSI, in comparison with contrast-enhanced magnetic resonance imaging (MRI), have the disadvantage of not being able to distinguish viable or hibernating myocardium from scar tissue among segments of non-contracting myocardium. Then, it is likely that the small number in some subgroups might have affected our capacity to uncover and characterize some of the associations, potentially leading to false negative results. Thus, the findings from the subgroup analysis require further validation. Finally, a small number of patients were not on standardized doses of medications; this further limits the generalizability of our results.

## 5. Conclusions

Persistent moderate or severe sMR is common (approximately 40%) in PCI patients. Elevated WMSI in CAD patients after PCI is a predictor of persistent moderate or severe sMR, adverse events in worsening HF, and long-term all-cause mortality. Given the adverse prognosis of persistent moderate or severe sMR, screening for WMSI in CAD patients with baseline moderate or severe sMR can yield important information that can be used to refine risk stratification for more intensive treatment based on established cardiovascular risk factors.

## Data Availability

Data are available from the corresponding author on reasonable request.

## Abbreviations

WMSI, wall motion score index; sMR, secondary mitral regurgitation; CAD, coronary artery disease; PCI, percutaneous coronary intervention; HF, heart failure; MACE, major adverse cardiovascular events; LVEF, left ventricular ejection fraction; ACS, acute coronary syndrome; AMI, acute myocardial infarction; CKD, chronic kidney disease; OR, odds ratio; HR, hazard ratio; CI, confidence interval.
